# TNF-TNFR1 Signaling Enhances the Protection Against *Neospora caninum* Infection

**DOI:** 10.3389/fcimb.2021.789398

**Published:** 2022-01-07

**Authors:** Flávia Batista Ferreira França, Murilo Vieira Silva, Mariana Ferreira Silva, Eliézer Lucas Pires Ramos, Vanessa dos Santos Miranda, Caroline Martins Mota, Fernanda Maria Santiago, José Roberto Mineo, Tiago Wilson Patriarca Mineo

**Affiliations:** Laboratory of Imunoparasitology “Dr. Mário Endsfeldz Camargo”, Department of Immunology, Institute of Biomedical Sciences, Federal University of Uberlândia, Uberlândia, Brazil

**Keywords:** neosporosis, TNF, chronic phase, effector molecules, antibodies

## Abstract

*Neospora caninum* is a protozoan associated with abortions in ruminants and neuromuscular disease in dogs. Classically, the immune response against apicomplexan parasites is characterized by the production of proinflammatory cytokines, such as IL-12, IFN-γ and TNF. TNF is mainly produced during the acute phases of the infections and binds to TNF receptor 1 (CD120a, p55, TNFR1) activating a variety of cells, hence playing an important role in the induction of the inflammatory process against diverse pathogens. Thus, in this study, we aimed to evaluate the role of TNF in cellular and humoral immune responses during *N. caninum* infection. For this purpose, we used a mouse model of infection based on wildtype (WT) and genetically deficient C57BL/6 mice in TNFR1 (*Tnfr1* ^-/-^). We observed that *Tnfr1* ^-/-^ mice presented higher mortality associated with inflammatory lesions and increased parasite burden in the brain after the infection with *N. caninum* tachyzoites. Moreover, *Tnfr1* ^-/-^ mice showed a reduction in nitric oxide (NO) levels *in vivo*. We also observed that *Tnfr1* ^-/-^ mice showed enhanced serum concentration of antigen-specific IgG2 subclass, while IgG1 production was significantly reduced compared to WT mice, suggesting that TNFR1 is required for regular IgG subclass production and antigen recognition. Based on our results, we conclude that the TNF-TNFR1 complex is crucial for mediating host resistance during the infection by *N. caninum*.

## Introduction

Neosporosis is an infectious disease caused by the parasite *Neospora caninum* which was first described as the causative of neurological disorders in dogs (Bjerkas et al., 1984). Formally classified in 1988, *N. caninum* is an obligatory intracellular parasite that belongs to Apicomplexa phylum (Dubey et al., 1988), a group composed by a range of parasites with great importance in human and veterinary medicine (Cowper et al., 2012). *N. caninum* is closely related to *Toxoplasma gondii*, presenting similar morphological characteristics (Dubey et al., 2007).

*N. caninum* has been reported to infect varied species of animals, including dogs, cattle, sheep, goat, among others. This infection occurs mainly through ingestion of food and/or water contaminated with oocysts eliminated in feces of canids, which are its definitive hosts (Almeria et al., 2017). Furthermore, the transplacental transmission is also considered an important transmission route, especially in cattle (Marugan-Hernandez, 2017), causing abortions and generating significant economic impacts in dairy and beef production (Dubey and Schares, 2011; Reichel et al., 2014; Mansilla et al., 2015).

The host immune response required to control *N. caninum* infection is based on the production of Th1-skewed inflammatory mediators. Moreover, the efficiency of Th1 adaptive responses against *N. caninum* is linked to the proper activation of innate immune cells, through parasite recognition by pattern recognition receptors (PRRs) such as Toll-Like Receptors (TLRs). Once activated, these receptors will signal through adapter molecules, as MyD88 or TRIF, which leads to production of inflammatory mediators as tumor necrosis factor (TNF) (Fereig and Nishikawa, 2020).

TNF is a cytokine that was originally described due to its antitumor properties (Carswell et al., 1975). Nowadays, it is known to be induced in response to injuries and infections, being produced mainly by macrophages, neutrophils, lymphocytes and other immune cells (Brietzke and Kapczinski, 2008; Davignon et al., 2018). Its function is linked to a variety of biological activities, including inflammation, cellular proliferation, differentiation, apoptosis and necroptosis (Aggarwal et al., 2012). Two distinct types of receptors – TNF receptor 1 (TNFR1) and TNF receptor 2 (TNFR2), may mediate TNF action (Wajant et al., 2003; Wajant and Siegmund, 2019). Its recognition by TNFR1 leads to the majority of its known biological activities, and is initiated by the activation of the transcription factor Nuclear Factor kappa-light-chain-enhancer of activated B cells (NF-κB) and mitogen-activated protein kinases (MAPK) (Chen and Goeddel, 2002; Wajant and Scheurich, 2011; Brenner et al., 2015).

Previous studies showed that this cytokine plays an important role in infections caused by protozoan parasites as *Toxoplasma*, *Leishmania* and *Trypanosoma* (Derouich-Guergour et al., 2001). For *N. caninum*, however, little is known about its role during the infection. It has been previously shown to reduce/inhibit parasite growth in primary tissue culture that used brain cells (Yamane et al., 2000; Jesus et al., 2013). In that sense, due to the lack of information regarding this key cytokine in the context of the infection, we aimed to evaluate the role of TNF in the regulation of cellular and humoral immune responses against *N. caninum*, using genetically deficient mice in its main receptor - TNF receptor I (TNFR1) - as a model.

## Materials and Methods

### Animals

Wild type C57BL/6 mice (WT, C57BL/6J, JAX 000664) and genetically deficient mice in TNF receptor superfamily member 1a (*Tnfr1* ^-/-^, C57BL/6J-*Tnfrsf1a^tm1lmx^*/J, JAX 003242, (Peschon et al., 1998), were bred and maintained under specific pathogen-free conditions at the institutional animal facility (REBIR/UFU), in an environment with controlled temperature (22-25°C), food and water *ad libitum* and light/dark cycles of 12h each. For the experiments described below, we used 6-8 weeks old female mice, housed in groups of up to 5 mice/cage. *Tnfr1* ^-/-^ mice were chosen due to the primordial role of the receptor in TNF signaling, rendering mice susceptible to other intracellular pathogens, although protecting against septic shock.

### Parasites and Antigen Preparation

Tachyzoites of the *N. caninum* isolate 1 (Nc-1) were maintained in monolayers of HeLa cells (CCL-2, ATCC, USA) at 37°C with 5% CO_2_ in RPMI 1640 medium supplemented with glutamine (2mM) and antibiotics/antimycotics (Thermo Scientific, USA). After cell lysis, the parasite suspensions were obtained as described previously (Davoli-Ferreira et al., 2016). The supernatant containing the parasite suspension was collected and centrifuged at 800 × g for 10 minutes at 4°C, and the pellet was resuspended in RPMI 1640. Tachyzoites were counted in a Neubauer chamber and used immediately for infection of mice or macrophages. The remainder of the parasites were washed twice (800 x g for 10 minutes at 4°C) with PBS and the final pellet was stored at -20°C for subsequent preparation of antigens.

*N. caninum* antigen lysate (NLA) was prepared according to the methods described previously (Mota et al., 2016). Parasite suspensions were diluted in PBS and treated with protease inhibitors (Complete Mini, Roche, Germany) and submitted to rapid freezing and thawing cycles, followed by sonication on ice. Parasite lysates were centrifuged (10,000×g, 30 min, 4°C), the resulting supernatant was collected and the protein concentration quantified using the Bradford method (Bradford, 1976). NLA aliquots were stored at -20°C until its use in ELISA procedures.

### Experimental Infections

Groups of WT and *Tnfr1* ^-/-^ mice (*n* = 10/group) were infected intraperitoneally (i.p.) with 1×10^7^ *N. caninum* tachyzoites for survival assays. For the analysis of acute and chronic phases of the infection, WT and *Tnfr1* ^-/-^ mice (*n* = 5/group) were infected with 1×10^6^ tachyzoites. All experiments were performed at least twice, in an independent manner, for confirmation purposes, and all sampled mice were analyzed individually. The acute phase experiments (7 days post-infection, dpi) were analyzed by the quantification of cytokines, parasite burden, histological alterations and nitric oxide production. During the chronic phase of the infection (30 dpi), parasite burden and histological changes were analyzed solely in brain tissues. For the quantification of specific IgG antibodies produced during the infection, serum of WT and *Tnfr1* ^-/-^ mice were collected at 0, 7, 14, 21 and 28 dpi.

### Determination of Parasite Burden

The parasite burden was determined in WT and *Tnfr1* ^-/-^ mice infected with 1 × 10^6^ tachyzoites. With that intent, tissue fragments and peritoneal cells were submitted to a real-time quantitative polymerase chain reaction (qPCR) using SYBR green detection system (Promega Co, USA), as previously described (Ribeiro et al., 2009), using primer pairs designed for the Nc5 sequence of *N. caninum*: Forward: 5’-GCTGAACACCGTATGTCGTAAA-3’; Reverse: 5’-AGAGGAATGCCACATAGAAGC- 3’. Genomic DNA was extracted from 20 mg of each analyzed tissue, or 1 × 10^6^ of peritoneal cells, using a commercial kit (Genomic DNA Kit, Promega), according to the manufacturer’s instructions. DNA concentrations were quantified by spectrophotometer (260/280 ratio; Nanodrop Lite, Thermo Scientific) and adjusted to 40 ng/µL with sterile DNAse free water. The reaction to determine parasite loads was performed in a Real-time PCR thermal cycler (StepOne Plus, Thermo Scientific, USA) and parasite counts were calculated by interpolation from a standard curve with known amounts of DNA extracted from *N. caninum* tachyzoites that were included in each analysis.

### Histological Analysis

Samples of livers, lungs, and brains of WT and *Tnfr1* ^-/-^ mice were collected and fixed in 10% buffered formalin at room temperature for 24 hours, and storage in alcohol 70% until the paraffin inclusion process. After inclusion, the organs were sliced (5μm thick) and deposited on microscopic slides, subsequently stained with hematoxylin and eosin for evaluation of inflammatory infiltrates and tissue damage (Mineo et al., 2009). The sections were photographed using an automated microscope (FSX100, Olympus, Japan).

### Cytokine Quantification

The production of the cytokines IL-12p40 and IFN-γ were measured in peritoneal fluids, sera, as well as liver and lung homogenates, using commercial ELISA kits, according to the protocols recommended by the manufacturer (BD Biosciences, USA). Tissue homogenates were prepared by grinding (IKA, Germany) 100 mg of freshly collected tissue in PBS supplemented with protease inhibitor cocktail (Complete Mini, Roche), followed by a centrifugation for supernatant removal and storage at -80°C until use (along with serum and peritoneal fluid samples). Optical density (OD) was read at 450 nm in a plate reader (M2e SpectraMax, Molecular Devices, USA). The concentration of each cytokine was determined by a standard curve with known concentrations of the cytokines expressed in pg/mL. Detection limits: IFN-γ = 4.1 pg/mL and IL-12p40 = 15.16 pg/mL. In addition, IFN-γ levels were also measured in serum samples using Cytometric Bead Array (CBA, BD Biosciences), read in a flow cytometer (FACSCanto II, BD Biosciences). IFN-γ CBA Detection limit: 0.5 pg/mL. IL-12 concentrations were measured in serum samples by ELISA method, as previously described.

### Quantification of Nitric Oxide

Nitric oxide (NO) production in peritoneal fluids of WT and *Tnfr1* ^-/-^ mice infected by *N. caninum* was determined through the reduction of nitrate and measurement of nitrite concentration by a commercial kit, according to the manufacturer’s instructions (R&D Systems Inc., USA). The assay was read at 540 nm (wavelength correction at 690 nm), and the concentration was estimated by a standard curve with lower detection limit at 0.78 µmol/L.

### Quantification of Specific IgG Antibodies

Serum levels of antigen specific IgG and its subclasses (IgG1 and IgG2) were measured in individual serum samples of infected WT and *Tnfr1* ^-/-^ mice by ELISA, as previously described (Mineo et al., 2010). Briefly, high-affinity microplates (Corning-Costar, USA) were coated with NLA (10 μg/mL) and incubated for 18 hours at 4°C. The reaction was blocked with 5% skim milk for total IgG and 1% bovine serum albumin (BSA) for IgG1 and IgG2a. Serum samples were diluted 1:25 and incubated for 1h (total IgG) and 2h (IgG1 and IgG2a) at 37°C. Peroxidase-labeled goat anti-mouse IgG (1:1000; Sigma-Aldrich Cat# A3673) or biotin-labeled goat anti-mouse IgG1 (1:4000; Caltag Lab/Invitrogen Cat# M32115) and anti-mouse IgG2a (1:2000; Caltag Lab/Invitrogen Cat # M32315) antibodies were added and incubated for 1h at 37°C. For detection of IgG1 and IgG2a, plates were further incubated with streptavidin-peroxidase (1:1000; Sigma-Aldrich), for 30 minutes at room temperature. Between each step, plates were washed with PBS plus 0.05% Tween 20 (PBS-T). The reaction was developed with 2,2-azino-bis-3-ethyl-benzthiazoline sulfonic acid (ABTS; KPL, USA) and the optical density (OD) determined at 405 nm in a plate reader (M2e SpectraMax, Molecular Devices).

For the immunoblots, we followed a previously published protocol (Ribeiro et al., 2009). Briefly, after NLA was submitted to electrophoresis in a 12% polyacrylamide gel under non-reducing conditions and transferred to nitrocellulose membranes, the reaction was blocked with 5% skim milk in PBS-T, incubated with individual mouse sera from each group, diluted 1:50, and then with peroxidase-goat anti-mouse IgG (diluted 1:1000) or biotin-labeled goat anti-mouse IgG1 (1:4000) and anti-mouse IgG2a (1:2000) antibodies. For the subclasses, an additional step with streptavidin-peroxidase (1:1000) was performed before the reaction was developed by adding 0.03% H_2_O^2^ and 3,3′-diaminobenzidine tetrahydrochloride (DAB; Sigma). The apparent molecular masses of antigenic bands were determined in relation to a standard molecular weight curve.

### Statistical Analysis

Statistical analyses were carried out using GraphPad Prism 9 software (GraphPad Software Inc., USA). Differences between groups were analyzed using Two-way ANOVA, with the respective Bonferroni post-tests, T-test, and Mann Whitney test, when appropriated. Survival rates were compared using Kaplan–Meier survival analysis, through a log-rank Mantel-Cox test. Values for *p* < 0.05 were considered significant.

## Results

### TNFR1 Contributes to Mice Survival During *N. caninum* Infection

**Figure 1:**
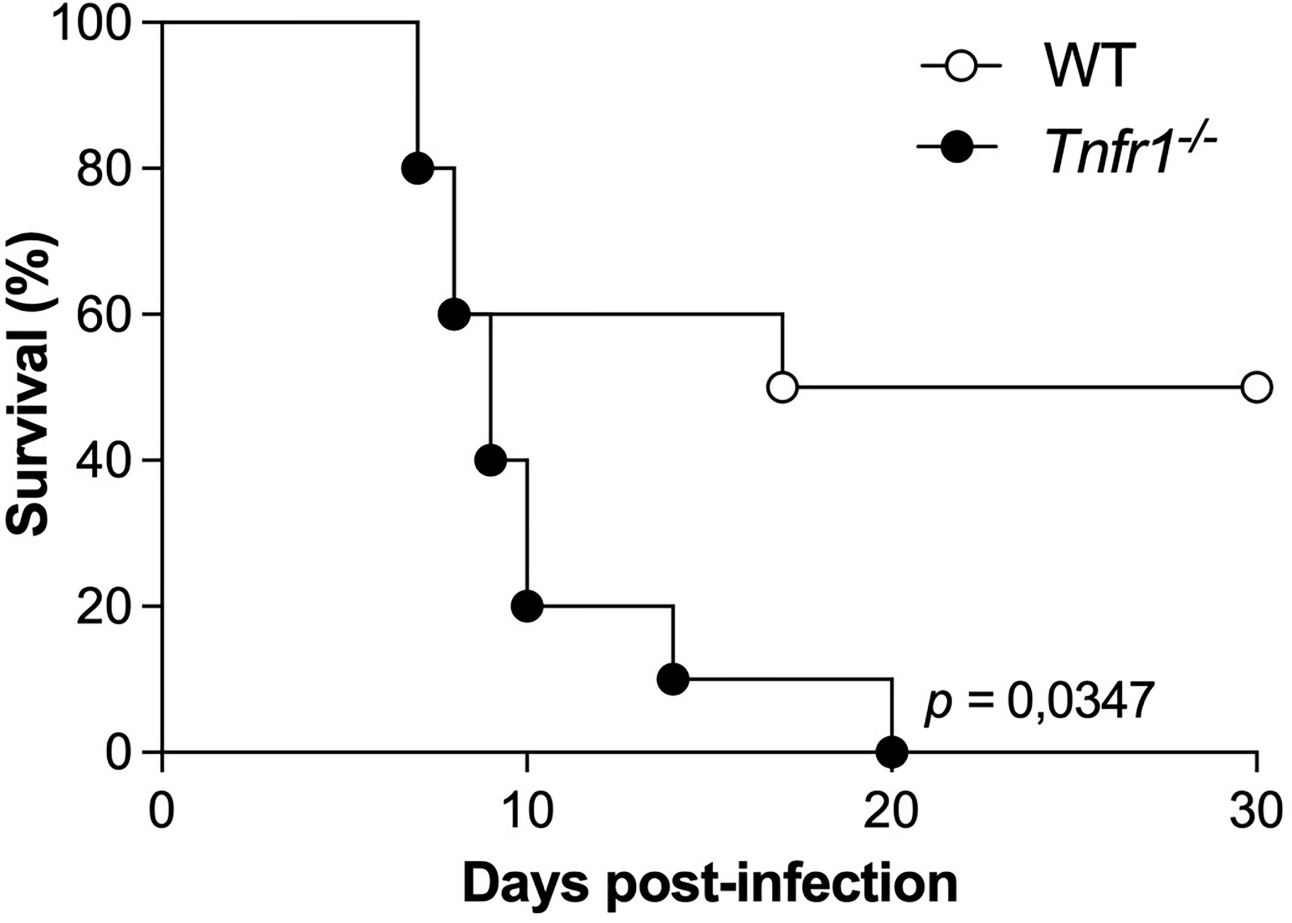
TNF is crucial for the survival of mice against *Neospora caninum*. Groups of WT and *Tnfr1* ^-/-^ mice were infected with 1×10^7^ Nc-1 tachyzoites and survival was observed for 30 days. Differences between groups were compared using Kaplan Meier survival analysis, through the log-rank (Mantel-Cox) test. Statistically significant differences (*p <* 0.05). Two independent experiments were performed with similar results, and the graphs represent the biological replicates (n = 10 mice/group) of one of those individual experiments.

To evaluate the importance of receptor 1 of TNF in the resistance of mice during the infection by *N. caninum*, WT and *Tnfr1* ^-/-^ mice were infected with a lethal dose of *N. caninum* for 50% of the animals (DL50, 1x10^7^ tachyzoites), and were monitored for 30 days for survival (**Figure 1**). We observed that mice of both experimental groups presented clinical signs around a week after the infection. However, *Tnfr1* ^-/-^ mice were less resistant to the infectious dose compared to WT, since 100% of the animals in this group had to be euthanized until the 20th day of infection, while – as expected – 50% of the WT mice survived (*P*<0.05).

### TNFR1 Affects Cerebral Parasite Load and Inflammation

**Figure 2:**
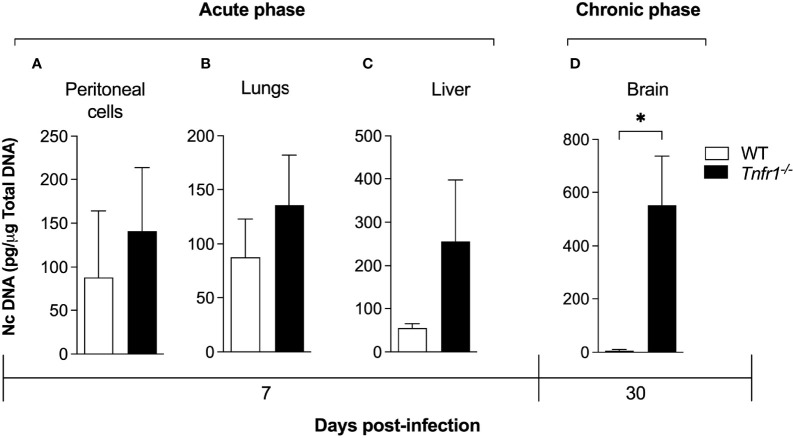
TNF is required to control the parasite burden during the chronic phase of infection. WT and Tnfr1-/- mice (n = 5 mice/group) infected with 1×106 Nc-1 tachyzoites were evaluated for parasite burden by qPCR. Peritoneal exudates **(A)**, lungs **(B)**, liver **(C)** and brains **(D)** were evaluated for the number of copies of the genomic sequence NC5. Two independent experiments were performed with similar results, and the graphs represent the biological replicates (n = 5 mice/group) of one of those individual experiments. Results are expressed as mean ± standard error of the mean (SEM). Data were analyzed using the Mann Whitney test. *Statistically significant differences (p < 0.05).

In order to evaluate whether the parasite burden profile was altered in the absence of TNFR1, we determined the amount of parasite genomic DNA in tissues of both groups infected with a sublethal dose of *N. caninum* tachyzoites (1x10^6^ parasites/mice). No significant difference was observed in parasite burden of peritoneal cells (**Figure 2A**), lungs (**Figure 2B**) and livers (**Figure 2C**) of *Tnfr1* ^-/-^ mice in relation to WT controls during the acute phase (7 days after infection). On the other hand, during the chronic phase (30 days after infection), quantification of the parasite burden in the brain showed increased quantities of *N. caninum* genomic DNA in *Tnfr1* ^-/-^ mice, if compared to the WT counterparts (*P*<0.05, **Figure 2D**).

**Figure 3:**
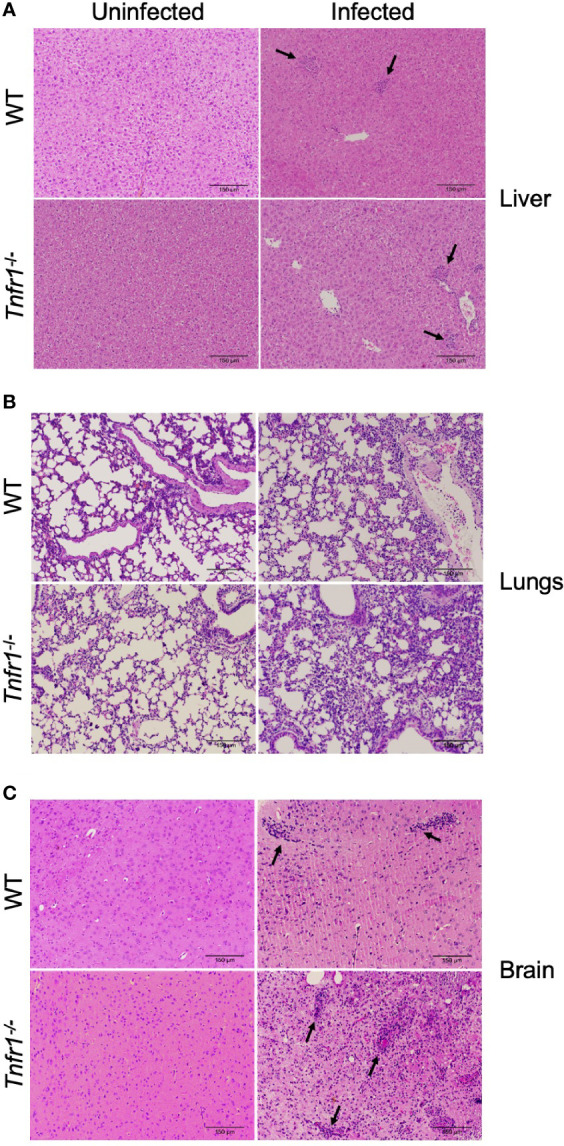
TNFR1 controls cerebral inflammation during chronification of *N. caninum* infection. Representative histological images of hepatic **(A)** and pulmonary **(B)** tissue sections analyzed 7 days post-infection, and brain sections **(C)** - collected at 30 days post-infection - in WT and *Tnfr1* ^-/-^ mice infected with 1 × 10^6^ tachyzoites of *N. caninum*. The histological sections were stained with H&E and analyzed under an optical microscope. Some focal inflammatory infiltrates are indicated by black arrows. Scale bar: 150µm. Two independent experiments were performed with similar results, and each figure represents the tissue of one biological replicate of one of those individual experiments. Tissues were obtained from 5 mice/group in each experiment.

We next analyzed whether TNF receptor 1 had a role in tissue inflammation during the infection with *N. caninum*. Inflammatory infiltrates were observed in all tissue sections obtained from both groups of mice (WT and *Tnfr1* ^-/-^). No clear differences were noted in liver and lung samples of both groups analyzed during the acute phase of infection (7 dpi). While sparse inflammatory foci were observed in liver sections (**Figure 3A**), a cellular infiltration in the pulmonary septum led to a marked loss of alveolar spaces in the lungs of both groups (**Figure 3B**). On the other hand, the absence of TNFR1 lead to an extensive inflammation in the brains of infected mice, with focal and diffuse infiltration of mononuclear cells throughout the parenchyma after 30 days of infection (**Figure 3C**), clearly compromising tissue integrity.

### NO Production Is Mediated by TNFR1 in Response to *N. caninum* Infection

**Figure 4:**
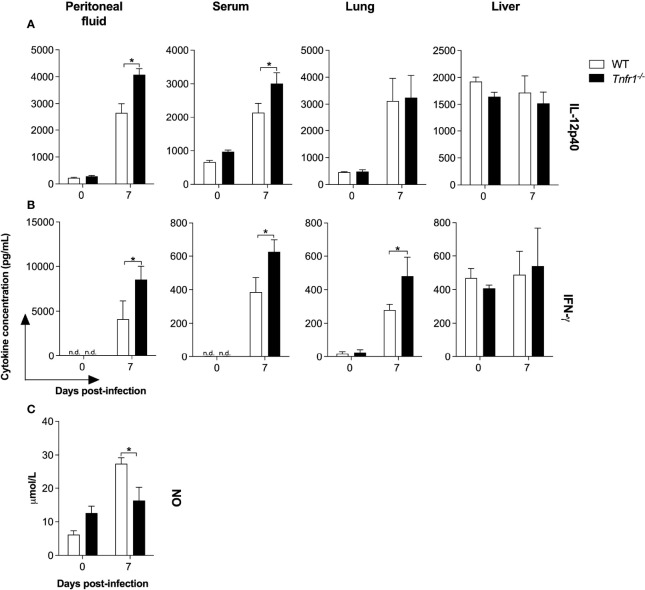
While TNFR1 does not downmodulate the production of key Th1 cytokines during the infection, it is essential for the production of proper levels of nitric oxide (NO). Groups of WT and *Tnfr1* ^-/-^ mice were evaluated for IL-12p40 **(A)** and IFN-γ **(B)** production 7 days after the infection with 1×10^6^ Nc-1 tachyzoites in the peritoneal fluids, serum samples, lung and liver homogenates. NO concentration was evaluated 7 days after infection in peritoneal fluids **(C)**. Values are expressed as mean ± standard error of the mean (SEM). Data were analyzed using the Two-way ANOVA test, followed by Bonferroni post-test. Differences were considered statistically significant (*) when *p* < 0.05. For the *in vivo* assays, two independent experiments were performed with similar results, and the graphs represent the biological replicates (n = 5 mice/group) of one of those individual experiments.

Based on the previous observations, we then went on to analyze whether the presence of TNF receptor 1 would modulate the production of key immune mediators. *In vivo*, we found an intriguing increment of IL-12 and IFN-γ levels in bodily fluids and lung homogenates of *Tnfr1* ^-/-^ mice during the first week of infection. IL-12p40 levels were significantly higher in the peritoneal exudates and serum samples of *Tnfr1* ^-/-^ mice compared to WT controls seven days post-infection (*P*<0.05, **Figure 4A**). In the same manner, IFN-γ production was also upregulated in the peritoneal fluid, serum and lung homogenates of *Tnfr1* ^-/-^ mice at the same time point (*P*<0.05, **Figure 4B**). No differences in the concentration of IL-12 and IFN-γ were detected in liver homogenates of both mouse lines, even between naïve and infected mice, which denotes a probable non-specific detection of the cytokines in that tissue. In order to explain these apparently contradictory results, we sought to determine whether the absence of TNF receptor 1 would impact the production of effector molecules known to be crucial for parasite killing. We analyzed the production of nitric oxide (NO) in the peritoneal fluids of WT and genetically deficient mice. Interestingly, the concentration of NO was significantly reduced in *Tnfr1* ^-/-^ mice if compared to WT controls (*P*<0.05, **Figure 4C**), suggesting that signaling through TNFR1 is necessary to induce the production of proper amounts of effector molecules in response to *N. caninum*.

### TNFR1 Alters the Balance of IgG Subclasses Raised Against *N. caninum*

**Figure 5:**
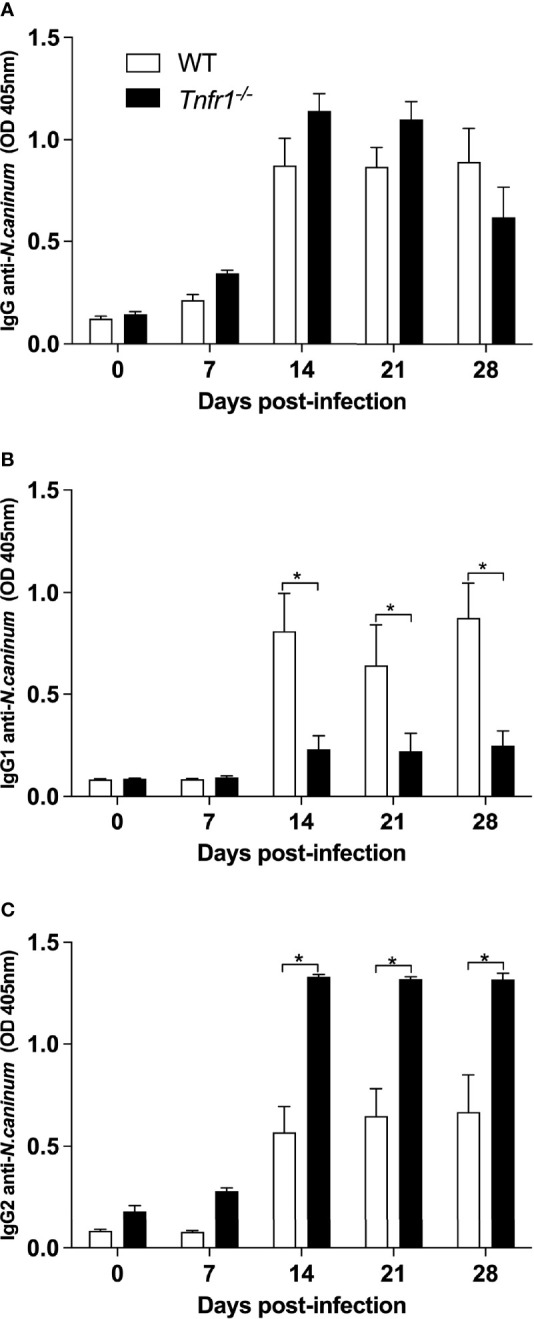
TNFR1 controls the production of IgG subclasses against soluble antigens. WT and *Tnfr1* ^-/-^ mice were infected intraperitoneally with 1×10^6^ Nc-1 tachyzoites and sampled weekly for serum samples until 28 days of infection. Levels of IgG **(A)**, IgG1 **(B)** and IgG2a **(C)** were determined by ELISA and values are indicated as optical density (OD) at 405nm, being expressed as mean ± standard error of the mean (SEM). Data were analyzed using the Two-way ANOVA test followed by Bonferroni post-test. Differences were considered statistically significant (*) when *p* < 0.05. Two independent experiments were performed with similar results, and the graphs represent the biological replicates (n = 5 mice/group) of one of those individual experiments.

We also aimed to elucidate the role of TNFR1 in the specific humoral immune response generated against *N. caninum*. With that intent, the concentration of *Neospora*-specific IgG and subclasses (IgG1 and IgG2) produced were measured weekly in the sera of infected WT and *Tnfr1* ^-/-^ mice against *N. caninum* soluble antigens. While no statistical differences were observed in the production of antigen-specific total IgG during the infection (**Figure 5A**), *Tnfr1* ^-/-^ mice produced significantly less antigen specific-IgG1 (*P*<0.05, **Figure 5B**) and higher concentration of IgG2 (*P*<0.05, **Figure 5C**) when compared to WT mice.

**Figure 6:**
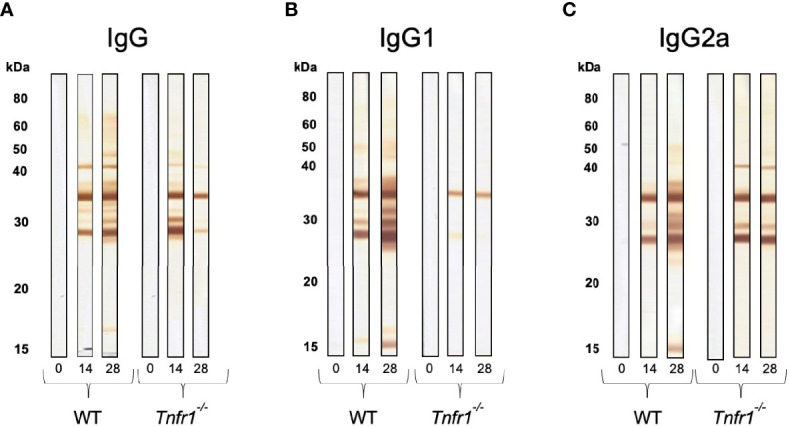
Immunoblots show the lack of proper antigen recognition in the absence of TNFR1. Individual serum samples of WT and *Tnfr1* ^-/-^ mice, obtained at 0, 14 and 28 days after the infection by *Neospora caninum*, were submitted to immunoblots for the determination of the pattern of recognition by specific IgG **(A)**, IgG1 **(B)** and IgG2a **(C)** antibodies against the parasite’s soluble antigen. We have displayed immunoblots that are representative of the reactivity obtained by an individual mouse/group/antibody, and that had similar results in two independent experiments with 5 mice/group each.

With the intention of verifying if antigen recognition was also altered by the absence of TNFR1, we ran western blots of the parasite’s soluble antigens against sera obtained from infected mice and checked for IgG reactivity. As expected, total IgG from WT mice recognized increasing numbers of parasite antigens as the infection progressed (**Figure 6A**), and this could also be observed for IgG1 and (**Figure 6B**) IgG2a (**Figure 6C**) subclasses. That same phenotype was not observed in *Tnfr1* ^-/-^ mice, which did not sustain a continuous increment in recognition of the soluble antigens by total IgG (**Figure 6A**). This was probably due to the failure of specific-IgG1 production and recognition of the antigens (**Figure 6B**), as IgG2 seemed to sustain a minimal recognition pattern of the antigens throughout the observed period, although recognizing less antigens at the latest time point (**Figure 6C**).

## Discussion

*Neospora caninum* has been associated with abortions in cattle and neuromuscular diseases in dogs, with a significant economic impact in countries that produce meat and dairy products (Reichel et al., 2014). Thus, to elucidate functions of immune modulators/activators is crucial for the development of prophylactic and therapeutic measures against neosporosis (Hemphill et al., 2016).

TNFR1 is the main receptor of TNF cytokine and is associated with the regulation of the inflammatory response, prevention of tissue damage, once it regulates the action of antigen presenting cells, and maintenance of tissue homeostasis (Zakharova and Ziegler, 2005; Hao et al., 2012; Wajant and Siegmund, 2019). Given the relevance of other pro-inflammatory cytokines in the immune response against *N. caninum*, we investigated here the role of TNF pathway in this infection. The importance of TNF in the resistance against other intracellular protozoa is well described (El-Sayed et al., 2016). For the closely related *T. gondii*, *Tnfr1/r2* ^-/-^ and *Tnfr1* ^-/-^ mice were highly susceptible to the infection, whereas *Tnfr2* ^-/-^ animals are resistant (Deckert-Schluter et al., 1998; Yap et al., 1998). In our study, infected *Tnfr1* ^-/-^ mice presented 100% of mortality during the course of infection, with high parasite burden and intense inflammatory infiltrates in the brain (30 d.p.i), indicating that this pathway contributes to host protection during infection latency. Also in agreement with what was found for *T. gondii*, previous studies demonstrated that *Tnfr2* ^-/-^ mice were resistant during the acute phase of infection by *N. caninum*, although mortality was described later on, during the chronic phase of infection (Ritter et al., 2002). The authors also described similar cerebral tissue damage amongst the groups, which is a clear difference from our results. In corroboration, the absence of intact TNF signaling did not alter the phenotype induced by the acute phase of the infection, with similar parasite burden and inflammatory status of livers and lungs of both groups of mice. Similar observations were previously recorded for lungs and brains of mice genetically deficient in the cytokine itself (*Tnf* ^-/-^) during the acute phase of the infection – no significant differences in parasite burden if compared to WT mice (Correia et al., 2015).

Cytokines as IL-12 and IFN-γ are important for the control of intracellular parasite replication and host resistance during the acute phase of the infection against *N. caninum* (Khan et al., 1997). As observed in this study, the absence of TNFR1 did not impair the production of these key cytokines. In fact, these cytokines were not only produced but also upregulated during the infection in peritoneum, serum, and lungs, probably in a compensatory mechanism related to the lack of effector molecules, as NO. TNF is a key component for the induction of these cellular effectors, due to the activation of STAT1 and NF-κB, in a positive feedback loop (Ohmori et al., 1997; Blaser et al., 2016). Also, the synergy and mutual dependence between TNF and IFN-γ to induce those crucial factors has also been intensively demonstrated (Nacy et al., 1991; Liew, 1993; Schäffer et al., 2006; Karki et al., 2021). In an infection model with *Citrobacter rodentium*, severe tissue damage and increased expression of IL-12p40 and IFN-γ in *Tnfr1* ^-/-^ mice was also observed (Gonçalves et al., 2001). On the other hand, WT and *Tnfr1* ^-/-^ mice showed similar production of IFN-γ during *T. gondii* infection, although *Tnfr1* ^-/-^ mice were highly susceptible to the infection (Deckert-Schluter et al., 1998). Partial IFN-γ signaling due to the lack of receptor 1 (IFN-γR1) had in TNF a compensatory mechanism to induce killing of *T. gondii*, while the same strategy would not be suficient to kill *Salmonella tiphymurium* (Janssen et al., 2002). In that sense, in a scenario where one of these key cytokines is missing, it’s likely that the lack of effector molecules will prompt parasite growth, and the increment in antigen availability will attract more inflammatory cells, in a recurrent phenomena that may lead the animals to death.

As speculated above, we wondered if TNF controlled *N. caninum* replication through the induction of effector molecules in the tested system. Previous work by our group demonstrated that nitric oxide is a key molecule for direct control of *N. caninum*, since deficient mice in iNOS presented uncontrolled parasite replication and extensive inflammatory lesions (Barros et al., 2019). Thus, as expected, the production of NO in *Tnfr1* ^-/-^ mice during *N. caninum* infection was diminished, indicating that both pathways are linked. Some studies also showed more susceptibility to *T. gondii* infection in *Tnf* ^-/-^ or *Tnfr1* ^-/-^ mice due to the absence of NO, which contributed to an increased parasite burden (Gazzinelli et al., 1993; Deckert-Schluter et al., 1998; Schluter et al., 2003). It is important to note that a study with murine microglial cells infected with *T. gondii* showed that TNF and NO production are crucial to control the infection even in the absence of IL-12 and IFN-γ (Pimenta et al., 2018).

The cytokine milieu induced by activated cells contribute to isotype changes of antibodies, where the profile of antibodies produced is directly related to the immune response triggered by the pathogen (Innes, 2007). Indeed, in *N. caninum* infection, the production of specific immunoglobulin is important to inhibit the invasion of tachyzoites in host cells and to control parasite replication (Aguado-Martinez et al., 2017). Our study showed that the absence of TNFR1 stimulated an exacerbated Th1 response, followed by a humoral response skewed towards IgG2a-specific antibodies. IFN-γ is a hallmark of Th1-type cellular responses against *N. caninum* (Mineo et al., 2010) and plays an important role in IgG class switch with predominant IgG2a isotype, inhibiting the expression of the other subclasses (Snapper and Paul, 1987; Long et al., 1998; Rojo-Montejo et al., 2009), specially IgG1 – an IgG subclass biased towards the Th2 phenotype (Stevens et al., 1988). IL-12 is a key cytokine in that context, committing CD4+ T cells to the Th1 phenotype (Germann et al., 1993). Overall, antigen specific IgG1 antibodies were not only less concentrated in the serum of *Tnfr1* ^-/-^ mice, but also did not recognize the major antigenic bands present in parasite’s soluble extract, denoting a probable loss of function that should be properly investigated. Also, detection of antigen-specific total IgG showed signs of early decay in *Tnfr1* ^-/-^ mice by ELISA and WB. Although we are not able to point out the specific reason for the phenomenon, it is probably related to the fundamental role of TNF signaling in B cell biology and its proper responses to antigens (Figgett et al., 2014).

Taken together, our results indicated that TNF - TNF receptor 1 signaling is crucial for an appropriated response against *N. caninum* infection, especially during chronification of the infection in the central nervous system. Since this pathway was shown to regulate the concentration of effector molecules and antibodies during the infection, future projects could evaluate the use of TNF as an immune modulator, aiming to reduce tissue inflammation and cerebral parasite burden in models of clinical neosporosis.

## Data Availability Statement

The original contributions presented in the study are included in the article/supplementary material. Further inquiries can be directed to the corresponding author.

## Ethics Statement

All experiments were previously approved by the ethics committee in animal experimentation (Comite de Ética na Utilização de Animais da Universidade Federal de Uberlândia - CEUA/UFU) under protocol 109/16. All procedures were carried out in accordance with the recommendations in the International Guiding Principles for Biomedical Research Involving Animals (https://olaw.nih.gov/sites/default/files/Guiding_Principles_2012.pdf), of the International Council for Laboratory Animal Science (ICLAS), countersigned by the Conselho Nacional de Controle de Experimentação Animal (CONCEA; Brazilian National Consul for the Control of Animal Experimentation). The institutional animal facility (Rede de Biotérios de Roedores—REBIR/UFU) is accredited by the National’s Commissions in Animal Experimentation (CONCEA, CIAEP:01.0105.2014) and Biosecurity (CTNBio, CQB 163/02).

## Author Contributions

Designed experiments, FF, FS, JM, and TM. Performed the experiments, FF, MVS, MFS, ER, VM, CM, and FS. Analyzed data and wrote the paper, FF and TM. Supplied reagents, JM and TM. All authors contributed to the article and approved the submitted version.

## Funding

This work was supported by Brazilian funding agencies (CNPq - 313761/2020-5; FAPEMIG - CVZ-PPM-00547-17, CVZ-APQ- 01313-14, RED-00313-16; CAPES-PrInt - AUXPE 2694/2018 - 88881.311510/2018-01). Funding sources had no involvement in study design, collection, analysis and interpretation of data, in writing the manuscript or in the decision to submit it for publication.

## Conflict of Interest

The authors declare that the research was conducted in the absence of any commercial or financial relationships that could be construed as a potential conflict of interest.

## Publisher’s Note

All claims expressed in this article are solely those of the authors and do not necessarily represent those of their affiliated organizations, or those of the publisher, the editors and the reviewers. Any product that may be evaluated in this article, or claim that may be made by its manufacturer, is not guaranteed or endorsed by the publisher.
